# Heme oxygenase-1 plays a pro-life role in experimental brain stem death via nitric oxide synthase I/protein kinase G signaling at rostral ventrolateral medulla

**DOI:** 10.1186/1423-0127-17-72

**Published:** 2010-09-07

**Authors:** Kuang-Yu Dai, Samuel HH Chan, Alice YW Chang

**Affiliations:** 1 Center for Translational Research in Biomedical Sciences, Chang Gung Memorial, Hospital-Kaohsiung Medical Center, Kaohsiung County 83301, Taiwan

## Abstract

**Background:**

Despite its clinical importance, a dearth of information exists on the cellular and molecular mechanisms that underpin brain stem death. A suitable neural substrate for mechanistic delineation on brain stem death resides in the rostral ventrolateral medulla (RVLM) because it is the origin of a life-and-death signal that sequentially increases (pro-life) and decreases (pro-death) to reflect the advancing central cardiovascular regulatory dysfunction during the progression towards brain stem death in critically ill patients. The present study evaluated the hypothesis that heme oxygnase-1 (HO-1) may play a pro-life role as an interposing signal between hypoxia-inducible factor-1 (HIF-1) and nitric oxide synthase I (NOS I)/protein kinase G (PKG) cascade in RVLM, which sustains central cardiovascular regulatory functions during brain stem death.

**Methods:**

We performed cardiovascular, pharmacological, biochemical and confocal microscopy experiments in conjunction with an experimental model of brain stem death that employed microinjection of the organophosphate insecticide mevinphos (Mev; 10 nmol) bilaterally into RVLM of adult male Sprague-Dawley rats.

**Results:**

Western blot analysis coupled with laser scanning confocal microscopy revealed that augmented HO-1 expression that was confined to the cytoplasm of RVLM neurons occurred preferentially during the pro-life phase of experimental brain stem death and was antagonized by immunoneutralization of HIF-1α or HIF-1β in RVLM. On the other hand, the cytoplasmic presence of HO-2 in RVLM neurons manifested insignificant changes during both phases. Furthermore, immunoneutralization of HO-1 or knockdown of *ho-1 *gene in RVLM blunted the augmented life-and-death signals exhibited during the pro-life phase. Those pretreatments also blocked the upregulated pro-life NOS I/PKG signaling without affecting the pro-death NOS II/peroxynitrite cascade in RVLM.

**Conclusions:**

We conclude that transcriptional upregulation of HO-1 on activation by HIF-1 in RVLM plays a preferential pro-life role by sustaining central cardiovascular regulatory functions during brain stem death via upregulation of NOS I/PKG signaling pathway. Our results further showed that the pro-dead NOS II/peroxynitrite cascade in RVLM is not included in this repertoire of cellular events.

## Background

The observation that asystole invariably takes place within hours or days after the diagnosis of brain stem death [1], the legal definition of death stipulated in professional or statutory documents from the United Kingdom [2,3], United States [4], European Union [5] or Taiwan [6], implies that permanent impairment of the brain stem cardiovascular regulatory machinery is intimately associated with this fatal phenomenon. It is therefore intriguing that based on power spectral analysis of systemic arterial pressure (SAP) signals from comatose intensive care unit patients [7-9], our laboratory found previously that a dramatic reduction or loss of the power density of the low-frequency (LF) component, which reflects dysfunction of central circulatory control, consistently occurs before brain stem death ensues. It follows that delineation of the cellular and molecular mechanisms that underpin the impending impairment of brain stem cardiovascular regulatory machinery should enrich the dearth of mechanistic information currently available on brain stem death. A logical neural substrate for this delineation resides in the rostral ventrolateral medulla (RVLM), which is long-known to be responsible for the maintenance of sympathetic vasomotor tone and stable SAP [10] and is the origin of the LF component [11] that presents itself as the life-and-death signal that disappears before brain stem death [12].

Mevinphos (3-(dimethoxyphosphinyl-oxyl)-2-butenoic acid methyl ester; Mev), a US Environmental Protection Agency Toxicity Category I pesticide, has been used in our laboratory as the experimental insult in an animal model for mechanistic evaluations of brain stem death [12-14] for two reasons. Systemic administration of Mev acts on RVLM to elicit cardiovascular toxicity [15]. More importantly, the distinct phases of an augmentation followed by a reduction of the LF power manifested during Mev intoxication resemble those exhibited by patients died of organophosphate poisoning during the progression towards brain stem death [9]. As such, they can be designated the pro-life and pro-death phase of cardiovascular regulation in this model of brain stem death [12]. Based on this model, our laboratory has previously reported that nitric oxide (NO) generated by NO synthase I (NOS I) in RVLM, followed by activation of the soluble guanylyl cyclase/cGMP/protein kinase G (PKG) cascade, is responsible for the pro-life phase; peroxynitrite formed by a reaction between NOS II-derived NO and superoxide anion underlies the pro-death phase [16,17]. On the other hand, NOS III in RVLM is not engaged in either the pro-life or pro-death phase of the Mev intoxication model of brain stem death [16].

Another pro-life program that our laboratory [13] identified in RVLM during brain stem death is heat shock protein 70 (HSP70), which ameliorates cardiovascular regulatory dysfunction via enhancing NOS I/PKG signaling and inhibiting NOS II/peroxynitrite cascade. We also showed previously that hypoxia-inducible factor-1 (HIF-1) acts as an upstream signal for HSP70 in RVLM during the pro-life phase of experimental brain stem death [18]. In addition, heme oxygenase-1 (HO-1) [19-21], and both NOS I [21,22] and NOS II [20,23] are known to be hypoxia responsive gene products; upregulation of HO-1 is mediated transcriptionally on HIF-1α activation [24,25].

A logical extension from those observations is that HO-1 may play a pro-life role in experimental brain stem death by interacting on one hand with HIF-1 and on the other with NOS I/PKG or NOS II/peroxynitrite signaling pathway in RVLM. The present study evaluated this hypothesis. Based on our Mev intoxication model, we demonstrated that on activation by HIF-1, HO-1 plays a preferential pro-life role in brain stem death by sustaining central cardiovascular regulatory functions via upregulation of NOS I/PKG signaling pathway in RVLM.

## Methods

All experimental procedures carried out in this study have been approved by the Laboratory Animal Committee of the Chang Gung Memorial Hospital-Kaohsiung Medical Center, and were in compliance with the guidelines for animal care set forth by this Committee.

### Animals

Adult male Sprague-Dawley rats (289 to 337 g, n = 316) purchased from the Experimental Animal Center of the National Science Council, Taiwan, Republic of China were used. Rats received preparatory surgery under an induction dose of pentobarbital sodium (50 mg kg^-1^, i.p.). During the experiment, animals received continuous intravenous infusion of propofol (20-25 mg kg^-1 ^h^-1^; Zeneca, Macclesfield, UK), which provided satisfactory anesthetic maintenance while preserving the capacity of central cardiovascular regulation [26]. They were allowed to breathe spontaneously with room air, and body temperature was maintained at 37°C by a heating pad.

### Mev intoxication model of brain stem death

SAP signals recorded from the femoral artery were subject to simultaneous on-line and real-time power spectral analysis [13-17,27], using a computer algorithm developed by our laboratory [28] that is specifically designed to deal with non-stationary signals encountered in clinical [7-9] and laboratory [13-17,27] settings. We were particularly interested in the LF (0.25-0.8 Hz) component of the SAP spectrum because it takes origin from RVLM [11] and the biphasic changes in LF power reflect the pro-life and pro-death phases during the progression towards brain stem death [12]. Heart rate (HR) was derived instantaneously from SAP signals. Since Mev induces comparable cardiovascular responses when given systemically or directly to RVLM [15], we routinely microinjected Mev bilaterally into RVLM to elicit site-specific effects [13-17,27]. Temporal changes in pulsatile SAP, mean SAP (MSAP), HR and power density of the LF component were routinely followed for 180 min after the administration of Mev, in an on-line and real-time manner.

### Microinjection of test agents

Microinjection bilaterally of test agents into the RVLM, each at a volume of 50 nl, was carried out stereotaxically and sequentially [13-17,27,29] via a glass micropipette connected to a 0.5-μl Hamilton microsyringe (Reno, NV, USA). The coordinates used were: 4.5-5 mm posterior to lambda, 1.8-2.1 mm lateral to midline, and 8.1-8.4 mm below the dorsal surface of cerebellum. Test agents employed included Mev (kindly provided by Huikwang Corporation, Tainan, Taiwan) and artificial cerebrospinal fluid (aCSF) that served as the vehicle control. A rabbit polyclonal antiserum against HIF-1α (Novus Biologicals, Littleton, CO, USA), HIF-1β (Lifespan Biosciences, Seattle, WA, USA), HIF-2α (Novus), HO-1 (Calbiochem, San Diego, CA, USA) or HO-2 (Santa Cruz, Santa Cruz, CA, USA) was used to affect immunoneutralization. As in previous studies [13,14], 0.02% Triton X-100 (Sigma-Aldrich, St. Louis, MO, USA) was added to facilitate transport of the antiserum across the cell membrane of RVLM neurons. Microinjection of normal rabbit serum (NRS; Sigma-Aldrich) plus 0.02% Triton X-100 served as the vehicle control. To avoid verbose presentation, however, the phrase 0.02% Triton X-100 is omitted from subsequent narration. Gene knockdown was executed using an antisense oligonucleotide (Quality Systems, Taipei, Taiwan) that targets against the coding region (base +10 to -9) of the *ho-1 *gene [30]: 5'-GGCGCTCCATCGCGGGACTG-3'; or the coding region (base +11 to -9) of the *ho-2 *gene [30]: 5'-TCTGAAGACATTGTTGCTGA-3'. The corresponding sense oligonucleotide: 5'-TCCAGCGGCGTCAGCGGTGC-3' (*ho-1*) or 5'-GATCTGACTTCAAGTGATTG-3' (*ho-2*) or scrambled oligonucleotide: 5'-CAGTCCCGCGATGGAGCGCC-3' (*ho-1*) or 5'-TCAGCAACAATGTCTTCAGA-3' (*ho-2*) was used as the control. The dose and treatment regimen were adopted from the literature that used the oligonucleotides for the same purpose as in the present study. To avoid the confounding effects of drug interactions, each animal received only one antiserum or oligonucleotide pretreatment.

### Collection of tissue samples

We routinely collected tissue samples [13,14,16,17] during the peak of the pro-life and pro-death phase (Mev group) or 30 or 180 min after microinjection of aCSF into RVLM (vehicle group). Animals were killed with an overdose of pentobarbital sodium, and tissues on both sides of the ventrolateral part of medulla oblongata, at the level of RVLM (0.5-2.5 mm rostral to the obex), were collected by micropunches made with a stainless steel bore (1 mm i.d.) and frozen in liquid nitrogen. Medullary tissues collected from anesthetized animals but without treatment served as the sham controls. Protein in the extracts was estimated by BCA Protein Assay (Pierce, Rockford, IL. USA).

### Protein expression

We employed Western blot analysis [13,14,16,17] to detect expression level of HO-1, HO-2, NOS I, PKG, NOS II or nitrotyrosine (marker for peroxynitrite) protein. The primary antiserum used for HIFs or HOs were the same as those used for immunoneutralization. The other primary antisera used included a rabbit polyclonal antiserum against NOS I (Santa Cruz), NOS II (Santa Cruz), PKG (Calbiochem); or a mouse monoclonal antiserum against nitrotyrosine (Upstate Biotechnology, Lake Placid, NY) or β-actin (Chemicon, Temecula, CA, USA). The secondary antisera used included horseradish peroxidase-conjugated donkey anti-rabbit IgG (Gehealthcare, Uppsala, Sweden) for HO-1, HO-2, NOS I, NOS II, PKG; or horseradish peroxidase-conjugated sheep anti-mouse IgG (Gehealthcare) for nitrotyrosine or β-actin. The amount of protein was quantified by the ImageMaster software (Amersham Pharmacia Biotech, Buckinghamshire, UK), and was expressed as the ratio relative to β-actin protein. Densitometric values that were not statistically different from the background were designated below detection limits.

### Immunofluorescence staining and confocal microscopy

We employed double immunofluorescence staining coupled with laser scanning confocal microscopy [13,14] to detect subcellular localization of HO-1 or HO-2 in RVLM neurons labeled with a mouse monoclonal antiserum against a specific neuron marker, neuron-specific nuclear protein (NeuN; Millipore, Billerica, MA, USA). Secondary antisera (Molecular Probes, Eugene, OR, USA) used included a goat anti-rabbit IgG conjugated with Alexa Fluor 568 for HO-1 or HO-2, and a goat anti-mouse IgG conjugated with Alexa Fluor 488 for NeuN. Tissues similarly processed but omitting primary antiserum against HO isoforms served as our negative controls. Immunoreactivity was viewed under a Fluorview FV1000 laser scanning confocal microscope (Olympus, Tokyo, Japan).

### Histology

In some animals that were not used for biochemical analysis, the brain stem was removed at the end of the physiological experiment and fixed in 30% sucrose in 10% formaldehyde-saline solution for at least 72 h. Frozen 25-μm sections of the medulla oblongata stained with neural red were used for histological verification of the microinjection sites.

### Statistical analysis

All values are expressed as the mean ± S.E.M. The effects of various treatments on the averaged value of MSAP or HR calculated every 20 min after administration of test agents or vehicle, the sum total of power density for LF component in the SAP spectra over 20 min, or the protein expression level in the ventrolateral medulla during each phase of Mev intoxication, were used for statistical analysis. One-way or 2-way ANOVA with repeated measures was used, as appropriate, to assess group means. This was followed by the Scheffé multiple-range test for post hoc assessment of individual means. P < 0.05 was considered to be statistically significant.

## Results

### Biphasic cardiovascular responses in experimental brain stem death

**Figure 1:**
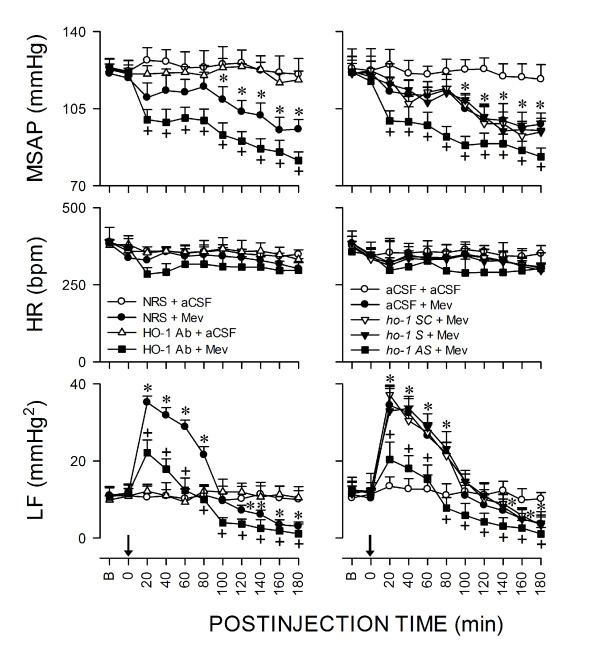
**Transcriptional upregulation of HO-1 in RVLM ameliorates failure of central cardiovascular regulation associated with experimental brain stem death**. Temporal changes in mean systemic arterial pressure (MSAP), heart rate (HR) or power density of the low-frequency (LF) component of SAP signals in rats that received pretreatment by microinjection bilaterally into RVLM of NRS (1:20) or HO-1 antiserum (1:20); or aCSF, scrambled (SC; 50 pmol), sense (S; 50 pmol) or antisense (AS; 50 pmol) *ho-1 *oligonucleotide (right column), 1 h or 24 h before local application (at arrow) of aCSF or Mev (10 nmol) to the bilateral RVLM. Values are mean ± SEM, n = 5-7 animals per experimental group. *P < 0.05 versus NRS+aCSF or aCSF+aCSF group, and ^+^*P *< 0.05 versus NRS+Mev or aCSF+Mev group at corresponding time-points in the Scheffé multiple-range test.

Figure 1 shows that co-microinjection bilaterally of Mev (10 nmol) and vehicle into RVLM elicited a progressive hypotension that became significant 100 min after application, accompanied by insignificant alterations in HR. Concurrent changes in power density of the LF component of SAP signals revealed two distinct phases of Mev-induced cardiovascular responses. The pro-life Phase I entailed a significantly augmented LF power that endured 80-100 min. The pro-death Phase II, which lasted the remainder of our 180-min observation period, exhibited further and significant reduction in the power density of this spectral component to below baseline to reflect failure of brain stem cardiovascular regulatory functions that precedes brain stem death [12].

### Preferential upregulation of HO-1 in RVLM during the pro-life phase

**Figure 2:**
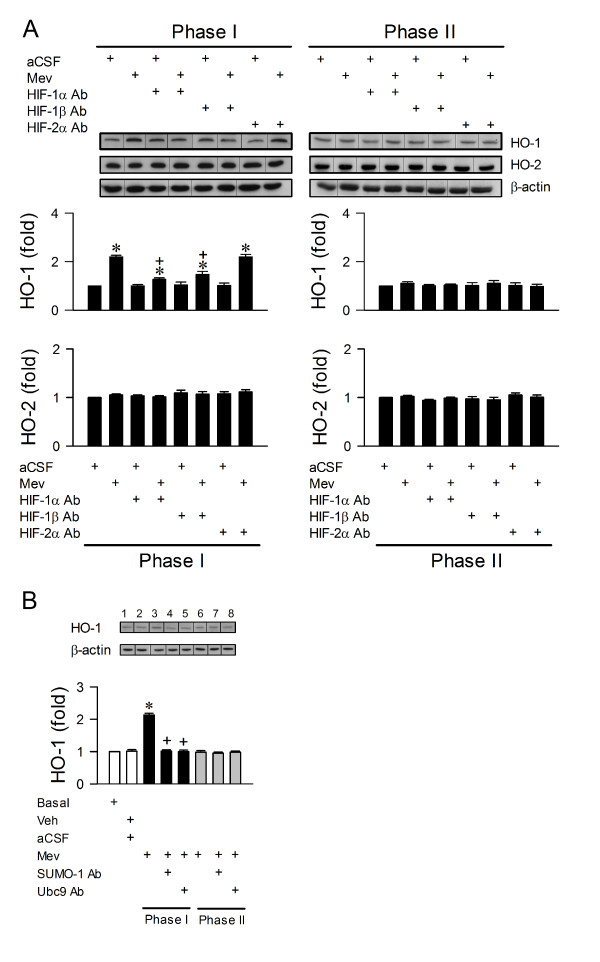
**Preferential transcriptional upregulation of HO-1 by HIF-1 in RVLM during the pro-life phase**. **A**. Illustrative gels or summary of fold changes against aCSF controls in ratio of HO-1 or HO-2 relative to β-actin protein detected in ventrolateral medulla of rats that received immunoneutralization of HIF-1α, HIF-1β or HIF-2α subunit in RVLM, 1 h before induction of Mev intoxication. **B**. Illustrative gels or summary of fold changes against aCSF controls of HO-1 expression detected in ventrolateral medulla of rats that received immunoneutralization of SUMO-1 or Ubc9, 1 h before induction of Mev intoxication. Values in **A **and **B **are mean ± SEM of triplicate analyses on samples pooled from 4-6 animals per experimental group. *P < 0.05 versus aCSF group and ^+^*P *< 0.05 versus Mev group in the Scheffé multiple-range test. Note that dividing lines are placed on the gel images to denote groupings of images from different parts of the same gel. Note also that numbers on top of the gels in **B **correspond to columns in the data summary.

The fundamental premise for HO-1 in RVLM to play a pro-life role in brain stem death is for it to be upregulated selectively during the pro-life phase in the Mev intoxication model. Our first series of experiments assessed this fundamental premise. Compared to aCSF controls, microinjection bilaterally of Mev (10 nmol) into RVLM significantly increased HO-1 protein expression (Fig. 2A) in ventrolateral medulla during Phase I, which returned to baseline during Phase II. On the other hand, HO-2 expression (Fig. 2A) remained relatively constant during both phases.

### Preferential upregulation of HO-1 in RVLM neurons during the pro-life phase

**Figure 3:**
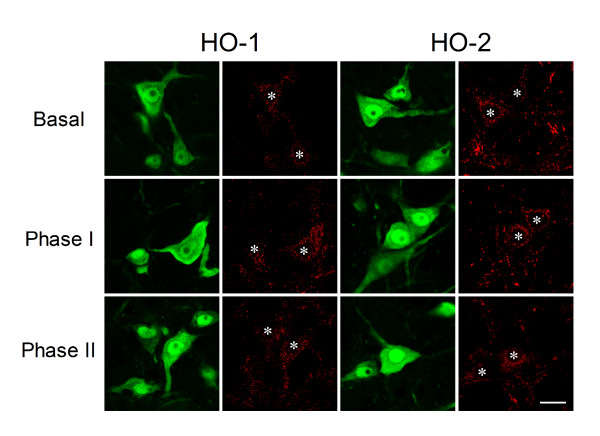
**Preferential upregulation of HO-1 in RVLM neurons during the pro-life phase**. Illustrative laser scanning confocal microscopic images showing cells in RVLM that were immunoreactive to NeuN (green fluorescence) and additionally stained positively for HO-1 or HO-2 isoform (red fluorescence) in sham controls (Basal) or during Phases I and II Mev intoxication. *Denotes location of nucleus in corresponding RVLM neuron. These results are typical of 4 animals from each experimental group. Scale bar, 10 μm.

Double immunofluorescence staining coupled with laser scanning confocal microscopy further revealed that the differential changes in HO-1 and HO-2 demonstrated in our biochemical analyses on protein extracts from ventrolateral medulla indeed took place at the neuronal level. Against a clearly defined nucleus and nucleolus in cells stained positively with the neuronal marker, neuron-specific nuclear protein (NeuN), the surge in HO-1 immunoreactivity during the pro-life phase was confined to the cytoplasm (Fig. 3), which subsided during Phase II. Again, the cytoplasmic presence of HO-2 in RVLM neurons exhibited indiscernible changes during both phases (Fig. 3).

### Preferential transcriptional upregulation of HO-1 by HIF-1 in RVLM during the pro-life phase

HO-1 is a well-known gene target that is regulated transcriptionally by HIF-1 [24,25]. Loss-of-function manipulation by immunoneutralization of HIF-1α or HIF-1β in RVLM significantly and selectively antagonized the Mev-induced augmentation of HO-1 protein expression in ventrolateral medulla during Phase I (Fig. 2A); anti-HIF-2α antiserum was ineffective in both phases (Fig. 2A). As an additional support for this observation, we extended results from a parallel study (unpublished data), which showed that augmented sumoylation of HIF-1α [31-34] is causally related to its enhanced stability or transcriptional activity in RVLM during the pro-life phase. Thus, immunoneutralization of SUMO-1 or Ubc9 (the only known conjugating enzyme for the sumoylation pathway) in RVLM (Fig. 2B) also significantly blunted the preferential upregulation of HO-1 in ventrolateral medulla during the pro-life phase.

### Activation of HO-1 in RVLM ameliorates failure of central cardiovascular regulation associated with experimental brain stem death

**Figure 4:**
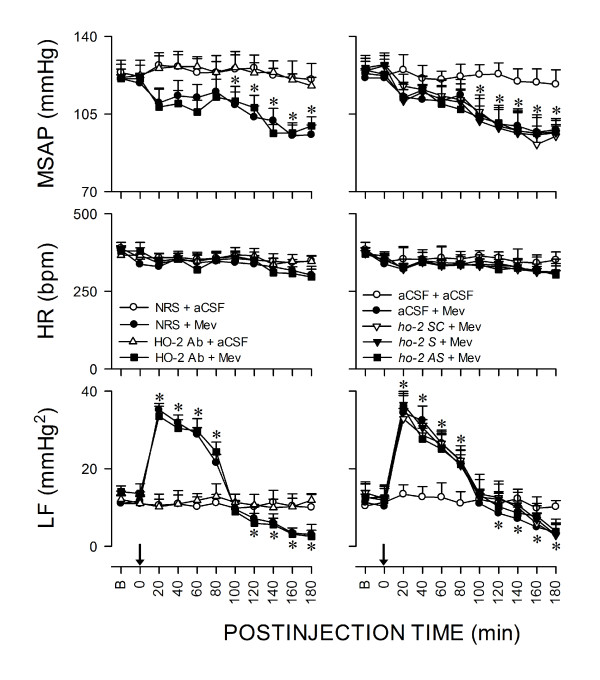
**Lack of effect of HO-2 in RVLM on failure of central cardiovascular regulation associated with experimental brain stem death**. Temporal changes in MSAP, HR or power density of the LF component of SAP signals in rats that received pretreatment by microinjection bilaterally into RVLM of normal rabbit serum (NRS; 1:20) or HO-2 antiserum (1:20); or aCSF, scrambled (SC; 50 pmol), sense (S; 50 pmol) or antisense (AS; 50 pmol) *ho-2 *oligonucleotide (right column), 1 h or 24 h before local application (at arrow) of aCSF or Mev (10 nmol) to the bilateral RVLM. Values are mean ± SEM, n = 5-7 animals per experimental group. *P < 0.05 versus NRS+aCSF or aCSF+aCSF group, and ^+^*P *< 0.05 versus NRS+Mev or aCSF+Mev group at corresponding time-points in the Scheffé multiple-range test.

We next employed immunoneutralization or gene-knockdown to establish that selective activation of HO-1 in RVLM is causally involved in central cardiovascular regulation during brain stem death. Pretreatment with microinjection bilaterally into RVLM of an anti-HO-1 antiserum or an antisense *ho-1 *oligonucleotide (Fig. 1), given 1 h or 24 h before local application of Mev (10 nmol), significantly and selectively potentiated the hypotension and antagonized the augmented LF power exhibited during Phase I; the hypotension and reduced LF power manifested during Phase II was further significantly enhanced. On the other hand, pretreatment with the same dose of anti-HO-2 (Fig. 4) antiserum, antisense oligonucleotide against *ho-2 *gene (Fig. 4), or sense or scrambled *ho-1 *(Fig. 1) or *ho-2 *(Fig. 4) oligonucleotide was ineffective against the phasic cardiovascular responses induced by Mev.

### Activation of HO-1 leads to phasic upregulation of NOS I/PKG signaling in RVLM

**Figure 5:**
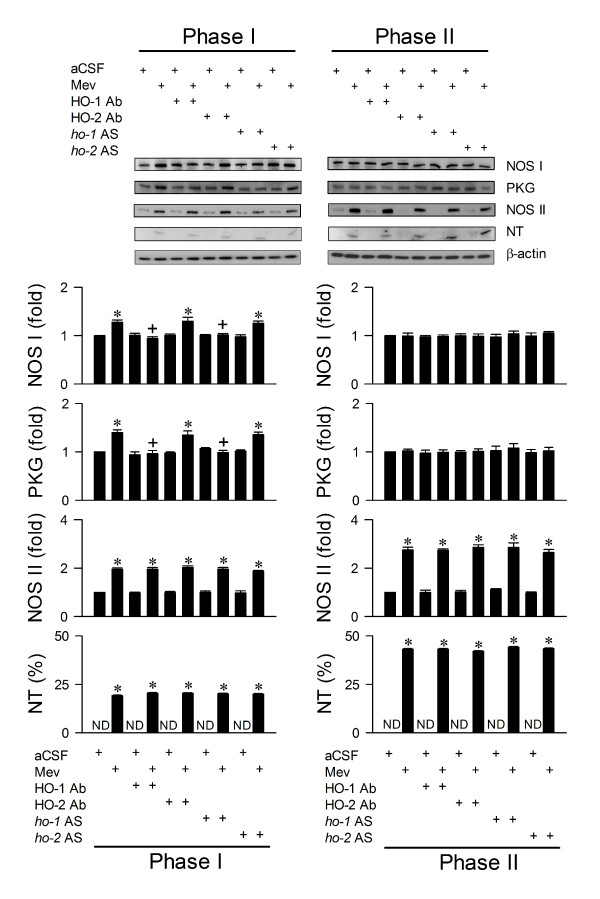
**Transcriptional activation of HO-1 leads to preferential upregulation of NOS I/PKG signaling in RVLM**. Illustrative gels or summary of fold changes against aCSF controls in ratio of nitric oxide synthase I (NOS I), protein kinase G (PKG), NOS II or nitrotyrosine (NT) relative to β-actin protein detected in ventrolateral medulla of rats that received immunoneutralization of HO-1 or HO-2, or knockdown of *ho-1 *or *ho-2 *gene in RVLM, 1 h or 24 h before induction of Mev intoxication. Note that NT is presented as % relative to β-actin because it is below detection limit (ND) in aCSF controls. Values are mean ± SEM of triplicate analyses on samples pooled from 4-6 animals per experimental group. *P < 0.05 versus aCSF group and ^+^*P *< 0.05 versus Mev group in the Scheffé multiple-range test.

**Figure 6:**
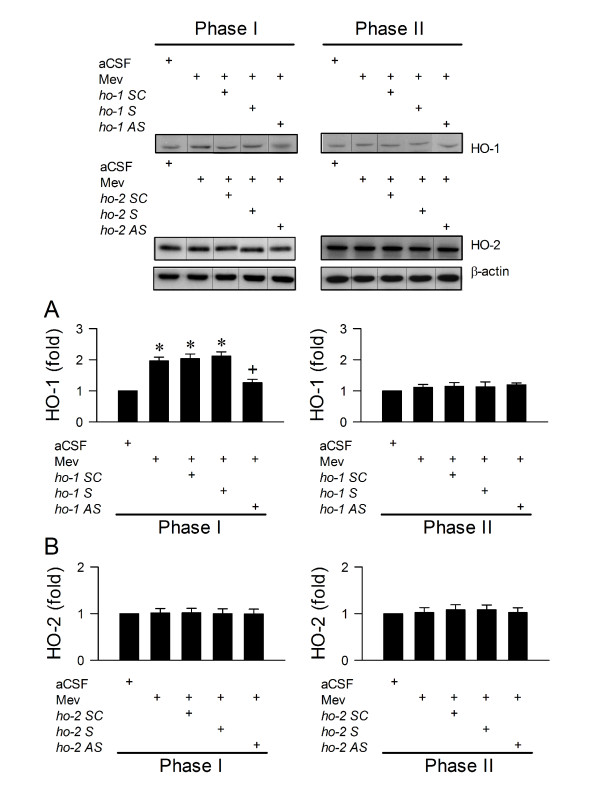
**Knockdown of *ho-1 *gene antagonized selectively the activation of HO-1 in RVLM**. Illustrative gels or summary of fold changes against aCSF controls in ratio of HO-1 (**A**) or HO-2 (**B**) relative to β-actin protein detected in ventrolateral medulla of rats that received knockdown of *ho-1 *or *ho-2 *gene in RVLM 24 h before induction of Mev intoxication. Values are mean ± SEM of triplicate analyses on samples pooled from 4-6 animals per experimental group. *P < 0.05 versus aCSF group and ^+^*P *< 0.05 versus Mev group in the Scheffé multiple-range test. Note that dividing lines are placed on the gel images to denote groupings of images from different parts of the same gel.

We demonstrated previously [13,14,16,17] that whereas NOS I/PKG signaling in RVLM is responsible for the pro-life phase, NOS II/peroxynitrite signaling underlies the pro-death phase of Mev intoxication. Our final series of experiments assessed whether HO-1 may subserve its pro-life role via modulation of these two signaling pathways. Immunoneutralization of HO-1 (Fig. 5) or knockdown of *ho-1 *gene (Fig. 5) blunted significantly and selectively the Mev-induced Phase I increase in NOS I or PKG protein expression in ventrolateral medulla. None of these pretreatments affected the progressive increase in NOS II or nitrotyrosine (marker for peroxynitrite) during both phases of Mev intoxication. Again, anti-HIF-2α or anti-HO-2 antiserum, antisense *ho-2 *oligonucleotide, or sense or scrambled *ho-1 *or *ho-2 *oligonucleotide was ineffective (Figs. 5 and 6) against the phasic Mev-induced NOS I, PKG, NOS II or nitrotyrosine protein expression in ventrolateral medulla.

### Effectiveness of gene knockdown

We also ascertained that our results from gene knockdown with antisense *ho-1 *oligonucleotide (Figs. 1 and 5) were accompanied by significant antagonism against the increase in HO-1 expression in ventrolateral medulla during Phase I Mev intoxication (Fig. 6), and sense or scrambled *ho-1 *oligonucleotide was ineffective. Likewise, the lack of alterations in HO-2 levels was not affected by pretreatment with antisense, sense or scrambled *ho-2 *(Fig. 6) oligonucleotide.

## Discussion

Based on a clinically relevant experimental model [12], we demonstrated that on transcriptional activation by HIF-1, HO-1 plays a preferential pro-life role during the progression towards brain stem death by sustaining central cardiovascular regulatory functions via upregulation of the NOS I/PKG signaling cascade in RVLM. We further showed that the engagement of HO-2 at RVLM in this process is minimal.

Both HO-1 and HO-2 are ubiquitously and catalytically active enzymes involved in the degradation of heme [35]. Whereas HO-2 is constitutively expressed under homeostatic conditions, HO-1 is an inducible isoform that is responsive to hypoxia or oxidative stress. As an antioxidant enzyme, HO-1 acts against oxidative stress by metabolizing heme to biliverdin, iron (Fe^2+^) and carbon monoxide [36]. It plays a neuroprotective role in mouse hippocampal neuron-derived HT22 cell line that is exposed to oxidative glutamate toxicity [37], or in homozygous HO-1 transgenic mice that are subject to middle cerebral artery occlusion [38]. Our laboratory reported previously [13] that severe tissue hypoxia, but not tissue hypo-perfusion, takes place in RVLM during Phase I Mev intoxication. It is therefore of interest that we found that activation of HIF-1 is causally related to the preferential upregulation of HO-1 in RVLM during the pro-life phase. HIF-1 is a heterodimer of two basic helix-loop-helix/PAS proteins, HIF-1α and HIF-1β [39]. Hypoxia stabilizes HIF-1α, and nucleus-bound translocation of the stabilized HIF-1α allows for formation of the HIF-1αβ heterodimer that becomes transcriptionally active [40]. The activated HIF-1αβ complex binds to target genes at hypoxia regulatory element (HRE), which contains the core recognition sequence 5'-RCGTG-3', leading to upregulation of hypoxia responsive gene products such as *ho-1 *[20]. Our results from loss-of-function manipulations of HIF-1α or HIF-1β showed that activation of HIF-1αβ complex leads to augmented HO-1 protein expression in RVLM neurons during the pro-life phase. Results from immunoneutralization of HO-1 protein or knockdown of *ho-1 *gene in RVLM further confirmed that this transcriptionally upregulated HO-1 is causally and preferentially related to sustaining central cardiovascular regulation during experimental brain stem death. On the other hand, our results indicated that whereas HO-2 is cytoprotective via phosphorylation by protein kinase C [41] and is present in RVLM neurons, it is minimally engaged in the cellular processes that underlie brain stem death.

Our results further lend credence to the notion that HO-1 acts as an intermediate between HIF-1 activation and the pro-life NOS I/PKG pathway in RVLM. NOS I [21,22] is known to be a hypoxia responsive gene product activated by HIF-1. Hypoxia increases NOS I expression that parallels activation of HIF-1α in piglet ventricular tissues [21]. An increase in NOS I mRNA and protein and HIF-1α protein expression also occurs in cerebral cortex of anemic rats [22]. The elevated NO level in hypoxic corpus callosum [42] or retina [43] is accompanied by an increase in mRNA and protein expression of HIF-1α and NOS I. Induction of HO-1 also rapidly restores NOS I expression in interstitial cells of Cajal and prevents oxidative stress in mice [44]. Of note is that the promoter region of *nos I *gene lacks HRE [45], the target site for HIF-1.Thus, it is of interest that by showing that immunoneutralization of HO-1 protein or knockdown of *ho-1 *gene blunted the surge of NOS I or PKG expression in RVLM during Phase I Mev intoxication, the present study demonstrated that HO-1 acts as the interposing signal between upregulation of HIF-1 and NOS I activation. Our laboratory showed previously that on activation by the HIF-1/HO-1 cascade [18], HSP70 ameliorates cardiovascular regulatory dysfunction during experimental brain stem death via enhancing NOS I/PKG signaling in RVLM [13]. It follows that the repertoire of cellular signals in RVLM during the pro-life phase of experimental brain stem death entails transcriptional upregulation of HO-1 by HIF-1, followed by activation of HSP70 that leads to sustained brain stem cardiovascular regulatory functions by the enhanced NOS I/PKG signaling.

Previous studies from our laboratory [16,17] demonstrated that the NOS II/peroxynitrite cascade in RVLM underlies central cardiovascular regulatory failure during the pro-death phase of experimental brain stem death. NOS II is also a well-known hypoxia responsive gene product [20,22]. Melillo *et al. *[46] showed that a sequence homologous to a hypoxia-responsive enhancer (NOS II-HRE) is responsible for activation of *nos II *gene in murine macrophages. A putative HIF-1 site (CTACGTGCT) in the murine NOS II gene was subsequently shown to be crucial for hypoxia-induced transcription in pulmonary artery endothelial cells [47] or cardiomyocytes [48]. Hypoxia-induced NOS II protein expression is transcriptionally upregulated by of HIF-1α in hippocampus of rats that received permanent middle cerebral artery occlusion [49]. However, since immunoneutralization of HO-1 protein and knockdown of *ho-1 *gene did not significantly affect the progressive augmentation of NOS II or nitrotyrosine levels in ventrolateral medulla during experimental brain stem death, the participation of HIF-1/HO-1 as cellular signals upstream to NOS II/peroxynitrite cascade is deemed minimal.

We recognize that the effectiveness of immunoneutralization depends on the specificity of the antiserum used. In this regard, the HIF-1α, HIF-1β, HIF-2α, HO-1 or HO-2 antiserum employed in the present study are all directed specifically against their respective antigens and do not cross-react with each other or other unrelated signaling systems. The same goes with the antisense oligonucleotides used to knock down *ho-1 *or *ho-2 *gene. The observations, for example, that immunoneutralization or gene knockdown of HO-1 exerted comparable effects on cardiovascular responses (Fig. 1) and NOS I/PKG signaling (Fig. 5), but not NOS II/peroxynitrite cascade (Fig. 5) during Mev intoxication further attest to the specificity of these treatments. The use of conditional knockout mouse model, which in essence is similar to antisense oligonucleotide treatment at RVLM, is another method of pretreatment. However, since our animal model of brain stem death is based on the rat, this approach was not adopted because of the concern for species difference.

## Conclusions

We conclude that transcriptional upregulation of HO-1 on activation of HIF-1 in RVLM plays a preferential pro-life role by sustaining cardiovascular regulatory functions during brain stem death via upregulation of NOS I/PKG signaling pathway. Our results further showed that NOS II/peroxynitrite signaling is not included in this repertoire of cellular events. This information should provide further insights on the etiology of brain stem death, and offer new directions for the development of therapeutic strategy against this fatal eventuality.

## Competing interests

The authors declare that they have no competing interests.

## Authors' contributions

KYD performed the cardiovascular experiments and carried out Western blot analysis and confocal microscopy. SHHC and AYWC conceived the study, participated in experimental design, and drafted and revised the manuscript. All authors have read and approved the final manuscript.

## References

[B1] Pallis C ABC of Brain Stem Death 1983 London: British Medical Journal Press 10.1136/bmj.286.6358.39 PMC1546672 6401455

[B2] Diagnosis of brain death. Statement issued by the honorary secretary of the Conference of Medical Royal Colleges and their Faculties in the United Kingdom on 11 October 1976 Br Med J 1976 2 1187 1188 10.1136/bmj.2.6045.1187 990836 PMC1689565

[B3] Diagnosis of death. Memorandum issued by the honorary secretary of the Conference of Medical Royal Colleges and their Faculties in the United Kingdom on 15 January 1979 Br Med J 1979 1 332 421104 10.1136/bmj.1.6159.332 PMC1597667

[B4] Report of the Medical Consultants on the Diagnosis of Death to the President's Commission for the Study of Ethical Problems in Medicine and Biomedical and Behavioral Research J Am Med Assoc 1981 246 2184 2186 10.1001/jama.246.19.2184 7289009

[B5] Haupt WF Rudolf J European brain death codes: a comparison of national guidelines J Neurol 1999 246 432 437 10.1007/s004150050378 10431766

[B6] Hung TP Chen ST Prognosis of deeply comatose patients on ventilators J Neurol Neurosurg Psychiatry 1995 58 75 80 10.1136/jnnp.58.1.75 7823073 PMC1073273

[B7] Kuo TBJ Yien HW Hseu SS Yang CCH Lin YY Lee LC Chan SHH Diminished vasomotor component of systemic arterial pressure signals and baroreflex in brain death Am J Physiol 1997 273 H1291 H1298 9321818 10.1152/ajpheart.1997.273.3.H1291

[B8] Yien HW Hseu SS Lee LC Kuo TBJ Lee TY Chan SHH Spectral analysis of systemic arterial pressure and heart rate signals as a prognostic tool for the prediction of patient outcome in intensive care unit Crit Care Med 1997 25 258 266 10.1097/00003246-199702000-00011 9034261

[B9] Yen DHT Yien HW Wang LM Lee CH Chan SHH Spectral analysis of systemic arterial pressure and heart rate signals of patients with acute respiratory failure induced by severe organophosphate poisoning Crit Care Med 2000 28 2805 2811 10.1097/00003246-200008000-00021 10966254

[B10] Spyer KM Central nervous mechanisms contributing to cardiovascular control J Physiol 1994 474 1 19 8014887 10.1113/jphysiol.1994.sp019997 PMC1160290

[B11] Kuo TBJ Yang CCH Chan SHH Selective activation of vasomotor component of SAP spectrum by nucleus reticularis ventrolateralis in rats Am J Physiol 1997 272 H485 H492 9038971 10.1152/ajpheart.1997.272.1.H485

[B12] Chan JYH Chang AYW Chan SHH New insights on brain stem death: From bedside to bench Prog Neurobiol 2005 77 396 425 10.1016/j.pneurobio.2005.11.004 16376477

[B13] Chan JYH Cheng HL Chou JLJ Li FCH Dai KY Chan SHH Chang AYW Heat shock protein 60 or 70 activates NOS 1- and inhibits NOS II-associated signaling, and depresses mitochondrial apoptotic cascade during brain stem death J Biol Chem 2007 282 4585 4600 10.1074/jbc.M603394200 17150954

[B14] Chan JYH Wu CHY Tsai CY Cheng HL Dai KY Chan SHH Chang AYW Transcriptional up-regulation of nitric oxide synthase II by nuclear factor-κB at rostral ventrolateral medulla in a rat mevinphos intoxication model of brain stem death J Physiol 2007 581 1293 1307 10.1113/jphysiol.2007.130872 17395621 PMC2170851

[B15] Yen DHT Yen JC Len WB Wang LM Lee CH Chan SHH Spectral changes in systemic arterial pressure signals during acute mevinphos intoxication in the rat Shock 2001 15 35 41 10.1097/00024382-200115010-00006 11198355

[B16] Chan JYH Chan SHH Chang AYW Differential contributions of NOS isoforms in the rostral ventrolateral medulla to cardiovascular responses associated with mevinphos intoxication in the rat Neuropharmacology 2004 46 1184 1194 10.1016/j.neuropharm.2004.01.017 15111025

[B17] Chan JYH Chan SHH Li FCH Cheng HL Chang AYW Phasic cardiovascular responses to mevinphos are mediated through differential activation of cGMP/PKG cascade and peroxynitrite via nitric oxide generated in the rat rostral ventrolateral medulla by NOS I and II isoforms Neuropharmacology 2005 48 161 172 10.1016/j.neuropharm.2004.08.012 15617736

[B18] Chang AYW Chan JYH Cheng HL Tsai CY Chan SHH Hypoxia-inducible factor 1/heme oxygenase 1 cascade as upstream signals in the prolife role of heat shock protein 70 at rostral ventrolateral medulla during experimental brain stem death Shock 2009 32 651 658 10.1097/SHK.0b013e3181a71027 19333137

[B19] Choi AM Alam J Heme oxygenase-1: function, regulation, and implication of a novel stress-inducible protein in oxidant-induced lung injury Am J Respir Cell Mol Biol 1996 15 9 19 8679227 10.1165/ajrcmb.15.1.8679227

[B20] Hellwig-Burgel T Stiehl DP Wagner AE Metzen E Jelkmann W Review: hypoxia-inducible factor-1 (HIF-1): a novel transcription factor in immune reactions J Interferon Cytokine Res 2005 25 297 310 10.1089/jir.2005.25.297 15957953

[B21] Louapre P Grongnet JF Tanguay RM David JC Effects of hypoxia on stress proteins in the piglet heart at birth Cell Stress Chaperones 2005 10 17 23 10.1379/CSC-74R.1 15832944 PMC1074566

[B22] McLaren AT Marsden PA Mazer CD Baker AJ Stewart DJ Tsui AKY Li X Yucel Y Robb M Boyd SR Liu E Yu J Hare GMT Increased expression of HIF-1α, nNOS, and VEGF in the cerebral cortex of anemic rats Am J Physiol Regulatory Integrative Comp Physiol 2007 292 R403 R414 10.1152/ajpregu.00403.2006 16973934

[B23] Natarajan R Jones DJ Fisher BJ Wallace TJ Ghosh S Fowler AA Hypoxia inducible factor-1: regulation by nitric oxide in posthypoxic microvascular endothelium Biochem Cell Biol 2005 83 597 607 10.1139/o05-047 16234848

[B24] Lee PJ Jiang BH Chin BY Iyer NV Alam J Semenza GL Choi AMK Hypoxia-inducible Factor-1 Mediates Transcriptional Activation of the Heme Oxygenase-1 Gene in Response to Hypoxia J Biol Chem 1997 272 5375 5381 10.1074/jbc.272.9.5375 9038135

[B25] Yang ZZ Zou AP Transcriptional regulation of heme oxygenases by HIF-1α in renal medullary interstitial cells Am J Physiol Renal Physiol 2001 281 900 908 10.1152/ajprenal.2001.281.5.F900 11592948

[B26] Yang CH Shyr MH Kuo TBJ Tan PPC Chan SHH Effects of propofol on nociceptive response and power spectra of electroencephalographic and systemic arterial pressure signals in the rat: correlation with plasma concentration J Pharmacol Exp Ther 1995 275 1568 1574 8531130

[B27] Yen DHT Chan JYH Huang CI Lee CH Chan SHH Chang AYW Coenzyme Q10 confers cardiovascular protection against acute mevinphos intoxication by ameliorating bioenergetic failure and hypoxia in the rostral ventrolateral medulla of the rat Shock 2005 23 353 359 10.1097/01.shk.0000156673.44063.e8 15803059

[B28] Kuo TBJ Chan SHH Continuous, on-line, real-time spectral analysis of systemic arterial pressure signals Am J Physiol Heart Circ Physiol 1993 264 H2208 H2213 10.1152/ajpheart.1993.264.6.H2208 8322952

[B29] Yen DHT Chan JYH Tsen HP Huang CI Lee CH Chan SHH Chang AYW Depression of mitochondrial respiratory enzyme activity in rostral ventrolateral medulla during acute mevinphos intoxication in the rat Shock 2004 21 358 363 10.1097/00024382-200404000-00011 15179137

[B30] Kaide JI Zhang F Wei Y Jiang H Yu C Wang WH Balazy M Abraham NG Nasjletti A Carbon monoxide of vascular origin attenuates the sensitivity of renal arterial vessels to vasoconstrictors J Clin Invest 2001 107 1163 1171 10.1172/JCI11218 11342580 PMC209275

[B31] Bae SH Jeong JW Park JA Kim SH Bae MK Choi SJ Kim KW Sumoylation increases HIF-1alpha stability and its transcriptional activity Biochem Biophys Res Commun 2004 324 394 400 10.1016/j.bbrc.2004.09.068 15465032

[B32] Berta MA Mazure N Hattab M Pouyssegur J Brahimi-Horn MC SUMOylation of hypoxia-inducible factor-1alpha reduces its transcriptional activity Biochem Biophys Res Commun 2007 360 646 652 10.1016/j.bbrc.2007.06.103 17610843

[B33] Carbia-Nagashima A Gerez J Perez-Castro C Paez-Pereda M Silberstein S Stalla GK Holsboer F Arzt E RSUME, a small RWD-containing protein, enhances SUMO conjugation and stabilizes HIF-1alpha during hypoxia Cell 2007 131 309 323 10.1016/j.cell.2007.07.044 17956732

[B34] Cheng J Kang X Zhang S Yeh ET SUMO-specific protease 1 is essential for stabilization of HIF1alpha during hypoxia Cell 2007 131 584 595 10.1016/j.cell.2007.08.045 17981124 PMC2128732

[B35] Shibahara S Han F Li B Takeda K Hypoxia and heme oxygenases: oxygen sensing and regulation of expression Antioxid Redox Signal 2007 9 2209 2225 10.1089/ars.2007.1784 17887916

[B36] Ryter SW Alam J Choi AMK Heme oxygenase-1/carbon monoxide: From basic science to therapeutic applications Physiol Rev 2006 86 583 650 10.1152/physrev.00011.2005 16601269

[B37] Satoh T Baba M Nakatsuka D Ishikawa Y Aburatani H Furuta K Ishikawa T Hatanaka H Suzuki M Watanabe Y Role of heme oxygenase-1 protein in the neuroprotective effects of cyclopentenone prostaglandin derivatives under oxidative stress Eur J Neurosci 2003 17 2249 2255 10.1046/j.1460-9568.2003.02688.x 12814358

[B38] Panahian N Yoshiura M Maines MD Overexpression of heme oxygenase-1 is neuroprotective in a model of permanent middle cerebral artery occlusion in transgenic mice J Neurochem 1999 72 1187 1203 10.1111/j.1471-4159.1999.721187.x 10037492

[B39] Wang GL Jiang BH Rue EA Semenza GL Hypoxia-inducible factor 1 is a basic-helix-loop-helix-PAS heterodimer regulated by cellular O2 tension Proc Natl Acad Sci USA 1995 92 5510 5514 10.1073/pnas.92.12.5510 7539918 PMC41725

[B40] Ema M Hirota K Mimura J Abe H Yodoi J Sogawa K Poellinger L Fujii-Kuriyama Y Molecular mechanisms of transcription activation by HLF and HIFα in response to hypoxia: their stabilization and redox signal-induced interaction with CBP/p300 EMBO J 1999 18 1905 1914 10.1093/emboj/18.7.1905 10202154 PMC1171276

[B41] Barañano DE Snyder SH Neural roles for heme oxygenase: contrasts to nitric oxide synthase Proc Natl Acad Sci USA 2001 98 10996 11002 10.1073/pnas.191351298 11572959 PMC58673

[B42] Kaur C Sivakumar V Ang LS Sundaresan A Hypoxic damage to the periventricular white matter in neonatal brain: role of vascular endothelial growth factor, nitric oxide and excitotoxicity J Neurochem 2006 98 1200 1216 10.1111/j.1471-4159.2006.03964.x 16787408

[B43] Kaur C Sivakumar A Foulds WS Luu CD Ling EA Cellular and Vascular Changes in the Retina of Neonatal Rats after an Acute Exposure to Hypoxia Invest Ophthalmol Vis Sci 2009 50 5364 5374 10.1167/iovs.09-3552 19474404

[B44] Choi KM Gibbons SJ Nguyen TV Stoltz GJ Lurken MS Ordog T Szurszewski JH Farrugia G Heme oxygenase-1 protects interstitial cells of Cajal from oxidative stress and reverses diabetic gastroparesis Gastroenterology 2008 135 2055 2064 10.1053/j.gastro.2008.09.003 18926825 PMC2796242

[B45] Jeong Y Won J Kim C Yim J 5'-Flanking sequence and promoter activity of the rabbit neuronal nitric oxide synthase (nNOS) gene Mol Cell 2000 10 566 574 10.1007/s10059-000-0566-7 11101149

[B46] Melillo G Musso T Sica A Taylor LS Cox GW Varesio L A hypoxia-responsive element mediates a novel pathway of activation of the inducible nitric oxide synthase promotor J Exp Med 1995 182 1683 1693 10.1084/jem.182.6.1683 7500013 PMC2192245

[B47] Palmer LA Semenza GL Stoler MH Johns RA Hypoxia induces type II NOS gene expression in pulmonary artery endothelial cells via HIF-1 Am J Physiol 1998 274 L212 L219 9486205 10.1152/ajplung.1998.274.2.L212

[B48] Jung F Palmer LA Zhou N Johns RA Hypoxic regulation of inducible nitric oxide synthase via hypoxia inducible factor-1 in cardiac myocytes Circ Res 2000 86 319 325 10679484 10.1161/01.res.86.3.319

[B49] Matrone C Pignataro G Molinaro P Irace C Scorziello A Di Renzo GF Annunziato L HIF-1alpha reveals a binding activity to the promoter of iNOS gene after permanent middle cerebral artery occlusion J Neurochem 2004 90 368 378 10.1111/j.1471-4159.2004.02483.x 15228594

